# Factors associated with job burnout among dental nurses: a cross-sectional study

**DOI:** 10.3389/fpubh.2026.1896451

**Published:** 2026-07-16

**Authors:** Chenxia Xiong, Rong Wang, Yingmei Li, Jinyu Yang, Lingli Pu, Yuesu Huang, Wenfang Liu, Ying Liu, Juan Liu, Xinchun Zou

**Affiliations:** 1 Yunnan Key Laboratory of Stomatology & Department of Oral and Maxillofacial Surgery, The Affiliated Stomatology Hospital, Kunming Medical University, Kunming, China; 2 Yunnan Key Laboratory of Stomatology & Department of the Second Clinic, The Affiliated Stomatology Hospital, Kunming Medical University, Kumming, China; 3 Yunnan Key Laboratory of Stomatology & Department of nursing, The Affiliated Stomatology Hospital, Kunming Medical University, Kumming, China; 4 Yunnan Key Laboratory of Stomatology & Department of the Chenggong Clinic, The Affiliated Stomatology Hospital, Kunming Medical University, Kumming, China; 5 Yunnan Key Laboratory of Stomatology & Department of Anesthesiology (Dental Operating Room), The Affiliated Stomatology Hospital, Kunming Medical University, Kumming, China; 6 Yunnan Key Laboratory of Stomatology & Department of Orthodontics, The Affiliated Stomatology Hospital, Kunming Medical University, Kumming, China; 7 Yunnan Key Laboratory of Stomatology & Department of Infection Management, The Affiliated Stomatology Hospital, School of Nursing, Kunming Medical University, Kunming, China; 8 Yunnan Key Laboratory of Stomatology & Department of General Office, The Affiliated Stomatology Hospital, Kunming Medical University, Kumming, China

**Keywords:** a cross-sectional study, dental nurses, depression, job burnout, occupational stress

## Abstract

**Background:**

Job burnout is a systemic challenge in healthcare, and dental nurses facing unique occupational stressors that linked to higher burnout symptom levels. Despite the growing recognition of burnout as a public health concern in health care workers, previous studies have paid relatively little attention to dental nurses.

**Objective:**

This study aimed to investigate the current status of job burnout among dental nurses and identify its associated factors.

**Methods:**

A cross-sectional study was conducted from September to October 2024, enrolling 261 dental nurses from a stomatological hospital in Yunnnan provinces of China. Data were collected using the Maslach Burnout Inventory-General Survey (MBI-GS), Nurse Job Stressor Scale (NJSS), Self-rating Depression Scale (SDS), Nurse's Career Identity Scale (NCIS), Social Support Rate Scale (SSRS), General Self-Efficacy Scale (GSES), and a self-designed general information sheet. A logistic regression model was constructed to analyse the associated factors correlated with job burnout.

**Results:**

The median score of job burnout was 38.67 (21.33–56.00), and the overall burnout prevalence was 33.7%. The results of logistic regression showed that depression [odds ratio (OR) = 1.117; 95% confidence interval (CI) (1.060, 1.177); *P* < 0.001], occupational stress [OR = 1.063; 95% CI (1.028, 1.098); *P* < 0.001] were associated factors of job burnout.

**Conclusion:**

This study explores job burnout in dental nurses and confirms depression and occupational stress as its associated factors. Attention to dental nurses' mental health and targeted interventions are critical to alleviating burnout and improving nursing quality.

## 1 Introduction

Dental nurses constitute an integral part of dental medical institutions. In addition to assisting dentists in performing four-handed dentistry techniques, they are also responsible for triage, health education, oral health care, infection control and other work (1). Their presence improves the efficiency of diagnosis and treatment, enhances the quality of medical care, boosts patient satisfaction, and effectively prevents cross-infection (2–4). However, the challenging professional environment, high-intensity workload, and significant occupational risks have led to the continuous increase of nurses' turnover intention. The State of the World's Nursing 2025 report reveals that the global nurse shortage continues to widen (5). This shortage is particularly pronounced in the oral health sector (6): demonstrated that the global density of oral health workforce (including dental nurses and assistants) is merely 5.31 per 10,000 population, with an inequitable distribution across countries and a limited skill mix that fails to meet the demands of the global oral disease burden. Consequently, the situation of dental nurses in China is also not optimistic. Relevant data indicate that the ratio of dentists to dental nurses in China generally fails to reach 1:1, making it difficult to carry out four-handed dentistry (7, 8). This grim situation jeopardizes the core role of dental nurses in improving oral healthcare quality and meeting the needs of socioeconomic and healthcare development. Retaining the existing dental nursing workforce and and attracting new talent to expand the dental nursing team, as well as supporting all dental nurses in providing high-quality healthcare services on an ongoing basis, poses a significant challenge to the healthcare system.

Job burnout is defined as a syndrome resulting from chronic workplace stress that has not been successfully managed. It is characterized by 3 dimensions: emotional exhaustion (EE), depersonalization (DP), and reduced personal accomplishment (PA) (9). Previous research has indicated that job burnout, which serves as a key factor influencing the turnover intention of nurses, not only results in reduced work efficiency and diminished work quality among nurses, but also triggers nurse-patient conflicts and contributes to impaired physical and mental health (10). In the past few years, the growing prevalence of burnout syndrome among health care workers has gained attention (11). A systematic review, which included 113 published studies, yielding a total of 45,539 nurses across 49 countries found that the pooled-prevalence rates was 11.23% for high burnout symptoms, indicating that one in ten nurses worldwide suffers from high burnout symptoms (12). However, previous studies have paid relatively little attention to dental nurses. Consequently, it is of great significance to assess the prevalence of job burnout among dental nurses, identify its associated factors, and take targeted measures. This can not only improve nursing quality and promote health, but also bring notable financial benefits to healthcare organizations, a study showed that a hospital's yearly expenditures linked to nurse turnover driven by burnout stood at US $16,736 per nurse. By contrast, this figure fell to US $11,592 per nurse annually at healthcare facilities that had implemented a burnout reduction initiative (13).

The job demands-resources (JD-R) model (14) which builds on the conceptualization of burnout proposing that factors associated with job burnout can generally be classified into two categories: job demands and job resources. Job demands refer to the psychosocial factors related to an individual's work that require continuous physical and mental exertion. They are “negative factors” in the workplace, such as occupational stress and depressive emotions. Job resources, by contrast, refer to all psychosocial and other factors related to an individual's work that are conducive to the achievement of work goals. They are “positive factors” in the workplace, such as career identity, social support, and self-efficacy. The model holds that job burnout develops along these two paths: (1) excessive job demands lead to exhaustion and (2) insufficient job resources lead to disengagement in the workplace (15). Thus, building on the theoretical perspective, this study incorporates above variables into the analytical framework to examine their associations with job burnout among dental nurses.

This study attempts to identify the factors associated with job burnout among dental nurses, thereby providing preliminary evidence to support dental healthcare managers and nursing administrators in implementing more targeted and timely occupational psychological interventions.

## 2 Method

### 2.1 Setting and participants

A cross-sectional study was conducted at a stomatological hospital in Yunnan, China. By convenient sampling, 261 dental nurses were recruited from September to October 2024. Nurses meeting the following criteria were invited to participate: (1) registered nurses, (2) informed consent to participate in this study. Exclusion criteria were: (1) internship and refresher nurses, and (2) sick leave, maternity leave, or leave of absence for more than 3 months.

### 2.2 Sample size

During the study design phase, we referred to the general rule of thumb (16) that the total sample size should be at least 10–15 times the number of independent variables to ensure model stability. Given that a maximum of 15 candidate variables were initially considered for univariate screening, we prospectively set a minimum recruitment target of 150 participants. Ultimately, we successfully enrolled 261 participants, exceeding this *a priori* target. For the final multivariable logistic regression model, 7 variables were retained. Using the more stringent “Events Per Variable (EPV)” criterion (17), which requires at least 10 events per covariate, the EPV for our final model was 88/7 = 12.6 (>10). Both criteria confirmed that the achieved sample size was more than adequate to provide reliable odds ratio estimates.

### 2.3 Ethical consideration

This study was approved by the Medical Ethics Committee of the Affiliated Stomatological Hospital of Kunming Medical University in August 2024 (Approval Number: KYKQ2024MEC0134). All participants signed an informed consent form, and nurses could refuse to fill in the questionnaire at any time if they did not want to participate, or withdraw from the questionnaire at any time if the content made them uncomfortable.

### 2.4 Measurement

#### 2.4.1 Socio-demographic information

A self-designed general information sheet was used to obtain nurses' socio-demographic and occupational data. Socio-demographic data included gender, age, marital status, monthly income, education, weekly exercise time, daily sleep time. Occupational data included length of employment, professional titles, weekly working hours.

#### 2.4.2 Job burnout

The Maslach Burnout Inventory-General Survey (MBI-GS) (18) was used in this study, it was developed by Maslach and consists of three dimensions: emotional exhaustion, depersonalization, and diminished personal accomplishment. With a total of 15 items, the MBI-GS was measured by a 7-point scale ranging from 0 (never) to 6 (every day). The total score ranges from 0 to 90, and higher scores indicate greater severity of job burnout. The original scale lacks uniform diagnostic cutoffs, a threshold of ≥50 was adopted in this study to dichotomize burnout status, following the methodology commonly applied in recent large-scale nursing studies (19, 20).

#### 2.4.3 Occupational stress

Occupational Stress was measured with the Nurse Job Stressor Scale (NJSS), which was developed by Li (21) based on the two most commonly used nurse job stressor scales internationally (22, 23). After being revised by nursing experts from the United States, Thailand and China and adjusted according to China's national conditions, a scale that is more suitable for nursing in China was thus formed. The NJSS includes 35 items and is divided into five dimensions: nursing profession and work, time and workload allocation, working environment and resources, patient care, and management and interpersonal relationships. This scale was measured by a 4-point Likert scale from 1 (no stress) to 4 (a lot of stress), and higher scores indicate greater stress. The overall Cronbach's α coefficient was 0.94 and ranged from 0.80 to 0.89 for each dimension.

#### 2.4.4 Depression

The Self-rating Depression Scale (SDS) (24) was used to evaluate depression level of dental nurses. The SDS included 20 items that measured mental, physical, and emotional symptoms and were rated by respondents in terms of how often the symptoms were experienced over the past week, using a 4-point scale ranging from 1 (none or very little) to 4 (most or all of the time). The raw score was calculated and converted into a standard score, with a total score of 100. The higher the SDS, the more serious of depression. The scale showed good internal consistency, the Cronbach's α coefficients was 0.81.

#### 2.4.5 Career identity

Career identity was measured with the Nurse's Career Identity Scale (NCIS), which was developed by the Nursing Management Teaching and Research Section of the University of Tokyo and translated into Chinese by Zhao (25). With a total of 21 items, the NCIS was measured by a 7-point Likert scale from 1 (Strongly disagree) to 7 (strongly agree). The total scores ranged from 21–147, with higher scores indicating a higher degree of identification with career. The Cronbach's α coefficient of this scale ranged from 0.69 to 0.84.

#### 2.4.6 Social support

The Social Support Rate Scale (SSRS) was used to measure the social support status, it was developed by Cauce et al. (26) in 1986, and has been widely used in China. The SSRS comprises 10 items, and were divided into three dimensions: objective social support, subjective social support, and the utilization of social support. Items' scores from each component of the SSRS were calculated. Items 1–4 and 8–10 are scored on a 4-point Likert scale, with each item rated from 1 to 4. Item 5 is scored from A to D, with each option assigned a score from 1 to 4. Items 6 and 7 are scored as 0 if the response is “no source,” while if there are multiple sources, each source is assigned a score. The higher the SSRS score, the higher level of social support. The Cronbach's alpha was 0.852.

#### 2.4.7 Self-efficacy

For self-efficacy, participants were evaluated using the General Self-Efficacy Scale (GSES) (27). It's a 4-point Likert scale ranging from 1 (incorrect) to 4 (completely correct), consisting of 10 items. The overall GSES score ranged between 10 and 40. A higher score indicated better self-efficacy. The scale showed good internal consistency with a Cronbach's α of 0.87.

### 2.5 Data collection

All dental nurses who satisfied the inclusion criteria were invited to participate in the study, after signing the informed consent form, nurses were asked to complete the questionnaire, all data were collected by the electronic questionnaire. The electronic questionnaire was first distributed to the head nurses, who then distributed the questionnaire to the nurses in the department and asked the clinical nurses who were willing to participate to fill it out. To ensure the quality of the filling, the questionnaire uses standardized instructions. All questions need to be answered and can only be answered once. After completing the questionnaires, the researcher will check the completion one by one and eliminate invalid questionnaires, such as those that are too short to fill in (< 5 min) and those with too regular answers.

### 2.6 Data analysis

All analyses were performed using R Statistical Software (Version 4.2.2, http://www.R-project.org, The R Foundation) and Free Statistics analysis platform (Version 2.2, Beijing, China). For all test, a two-sided *P*-value < 0.05 was considered statistically significant. For measurement data adhering to a normal distribution, descriptions were presented using mean values and standard deviations; in cases of non-normal distribution, the median and interquartile range were applied instead. Count data, meanwhile, were characterized by frequencies and constituent ratios. Subsequently, differences between dental nurses with and without job burnout were assessed using *t*-tests, Mann–Whitney *U* tests, or Chi-square tests. Candidate variables with *P* < 0.05 in the univariate analyses were entered into a binary logistic regression model with job burnout as the outcome. The model's goodness-of-fit was assessed with the Hosmer–Lemeshow test. Finally, to explore potential non-linear dose-response relationships beyond the standard logistic regression assumptions, we conducted two exploratory *post-hoc* visualization analyses on the variable with the strongest effect size identified in the final model: (1) restricted cubic spline (RCS) regression with 3 knots (at the 10th, 50th, and 90th percentiles) was used to flexibly model and visualize the exposure-outcome relationship; (2) boxplots were generated to illustrate the distribution of the outcome across different exposure subgroups, with intergroup comparisons performed via the Mann–Whitney *U* test. These analyses were hypothesis-generating rather than confirmatory.

## 3 Result

### 3.1 Socio-demographic information

Of the 269 dental nurses who received the electronic questionnaire, 263 completed the survey. A total of 261 valid questionnaires were selected after one-by-one inspection (2 questionnaires were excluded due to short response time). The average age was 31.05 years, the most were female (96.6%) and had a bachelor's degree (89.3%). More than half of the participants were married, with no more than 1.5 h of exercise per week and < 7 h of sleep per day. The average length of employment was 7.34 years, most had only entry-level titles and worked more than 40 h per week. The results are shown in Table 1.

**Table 1:** Sample characteristics and correlations of demographic factors, occupational stress, depression, career identity, social support, self-efficacy with job burnout (*N* = 261).

Variable		Total (*n* = 261)	No burnout (*n* = 173)	Burnout (*n* = 88)	χ^2^/*Z*/*t*	*P*
		*n* (%)	*n* (%)	*n* (%)		
Gender	Male	9 (3.4)	7 (4.0)	2 (2.3)	χ^2^ = 0.551	0.458
	Female	252 (96.6)	166 (96.0)	86 (97.7)		
Age (years)	20–29	135 (51.7)	96 (55.5)	39 (44.3)	χ^2^ = 6.584	0.037
	30–39	97 (37.2)	55 (31.8)	42 (47.7)		
	≥40	29 (11.1)	22 (12.7)	7 (8.0)		
Marital status	Single/divorced	124 (47.5)	78 (45.1)	46 (52.3)	χ^2^ = 1.208	0.272
	Married	137 (52.5)	95 (54.9)	42 (47.7)		
Monthly income (yuan)	≤ 5,000	46 (17.6)	27 (15.6)	19 (21.6)	χ^2^ = 1.930	0.381
	5,001–8,000	183 (70.1)	126 (72.8)	57 (64.8)		
	>8,000	32 (12.3)	20 (11.6)	12 (13.6)		
Education	Associate degree	23 (8.8)	17 (9.8)	6 (6.8)	χ^2^ = 0.657	0.418
	Bachelor's degree and above	238 (91.2)	156 (90.2)	82 (92.2)		
Weekly exercise time (hours)	<150	165 (63.2)	99 (57.2)	66 (75.0)	χ^2^ = 9.245	0.010
	150–300	73 (28.0)	54 (31.2)	19 (21.6)		
	>300	23 (8.8)	20 (11.6)	3 (3.4)		
Daily sleep time (hours)	≤ 7	194	123 (71.1)	71 (80.7)	χ^2^ = 2.808	0.094
	>7	67	50 (28.9)	17 (19.3)		
Length of employment (years)	<5	165 (33.3)	59 (34.1)	28 (31.8)	χ^2^ = 0.335	0.846
	5–10	73 (46.4)	78 (45.1)	43 (48.9)		
	>10	23 (20.3)	36 (20.8)	17 (19.3)		
Professional titles	Registered nurse	56 (21.5)	44 (25.4)	12 (13.6)	χ^2^ = 5.069	0.079
	Senior registered nurse	125 (47.9)	77 (44.5)	48 (54.5)		
	Supervisor nurse and above	80 (30.6)	52 (30.1)	28 (31.8)		
Weekly working hours	<40	26 (10.0)	18 (10.4)	8 (9.1)	χ^2^ = 5.719	0.057
	40–50	156 (59.8)	111 (64.2)	45 (51.1)		
	>50	79 (30.2)	44 (25.4)	35 (39.8)		
Occupational stress, mean (SD)		86.90 (14.94)	81.99 (13.55)	96.55 (12.73)	*t* = −8.373	<0.001
Depression, mean (SD)		50.55 (10.90)	46.18 (8.41)	59.13 (10.12)	*t* = −10.970	<0.001
Career identity, median (IQR)		118.00 (106.00–126.00)	122.00 (113.00–130.00)	106.00 (99.00–116.00)	*Z* = 6.634	<0.001
Social support, mean (SD)		41.28 (8.59)	43.20 (8.02)	37.50 (8.46)	*t* = 5.332	<0.001
Self-efficacy, mean (SD)		26.73 (5.99)	28.16 (5.41)	23.95 (6.11)	*t* = 5.673	<0.001

### 3.2 Level of job burnout and its associated factors

Normal Q–Q Plot and histogram showed that job burnout and career identity were not normally distributed, so they were described by median and interquartile range, as shown in Table 1. The median score of job burnout was 38.67 (21.33–56.00), and 33.7% of the participants had job burnout, among whom 12 had severe burnout (the score of MBI-GS is >75).

### 3.3 Univariate analysis of job burnout among dental nurses

Univariate analysis was used to compare the differences in potential associated factors between participants with and without job burnout, as shown in Table 1. The relationship between socio-demographic variables and job burnout was performed by chi-square test. As for other associated factors, since career identity did not show a normal distribution, the Mann–Whitney *U* test was used, and *t*-test was used for the rest. The results are illustrated in Table 1. The findings showed that job burnout was associated with age, weekly exercise time, occupational stress, depression, career identity, social support, and self-efficacy.

### 3.4 Analysis of associated factors of job burnout among dental nurses

Table 2 showed the results of binary logistic regression analysis by entering the statistically significant variables from univariate analysis. The results showed that depression [odds ratio (OR) = 1.117; 95% confidence interval (CI) (1.060, 1.177); *P* < 0.001], occupational stress [OR = 1.063; 95% CI (1.028, 1.098); *P* < 0.001] were independent associated factors of job burnout, indicating that dental nurses with higher occupational stress or depressive symptoms had a higher likelihood of experiencing job burnout.

**Table 2:** Binary logistic regression analysis of factors associated with job burnout among dental nurses (*N* = 261).

Independent variable		*B*	SE	OR	95% CI for OR		*P*	VIF
					Lower	Upper		
Constant		−5.801	2.680	0.003	–	–	0.030	–
Age	20–29	Ref						3.321
	30–39	0.760	0.410	2.139	0.958	4.773	0.063	
	≥40	0.183	0.631	1.201	0.349	4.134	0.771	
Weekly exercise time	< 150	Ref						1.077
	150–300	−0.005	0.426	0.995	0.432	2.292	0.991	
	>300	−0.249	0.849	0.779	0.147	4.119	0.769	
Occupational stress		0.061	0.017	1.063	1.028	1.098	< 0.001	1.297
Depression		0.111	0.027	1.117	1.060	1.177	< 0.001	1.405
Career identity		−0.030	0.016	0.971	0.941	1.001	0.061	1.471
Social support		−0.039	0.025	0.962	0.916	1.010	0.116	1.542
Self-efficacy		−0.055	0.037	0.947	0.880	1.018	0.142	1.354

The Hosmer–Lemeshow test was used to assess the goodness-of-fit of the model. The results showed that χ^2^ = 6.583, *P* = 0.582, suggesting no significant difference between the fitted values and actual observed values of the model, indicating that the binary Logistic regression model had a good fit.

Additionally, we assessed multicollinearity among the independent variables included in the final model by calculating the variance inflation factor (VIF). As shown in Table 2, The VIF values of all included variables ranged from 1.077 to 3.321 (all < 5), indicating no significant multicollinearity in the regression model.

### 3.5 Additional exploratory analyses for depression

Among all significant covariates in the final logistic regression model, depression exhibited the largest magnitude of association with job burnout [OR = 1.117, 95% CI: (1.060–1.177), *P* < 0.001]. To further characterize this relationship, we performed the two exploratory visualization analyses described in the Methods.

#### 3.5.1 Dose-Response relationship between depression and job burnout

**Figure 1:**
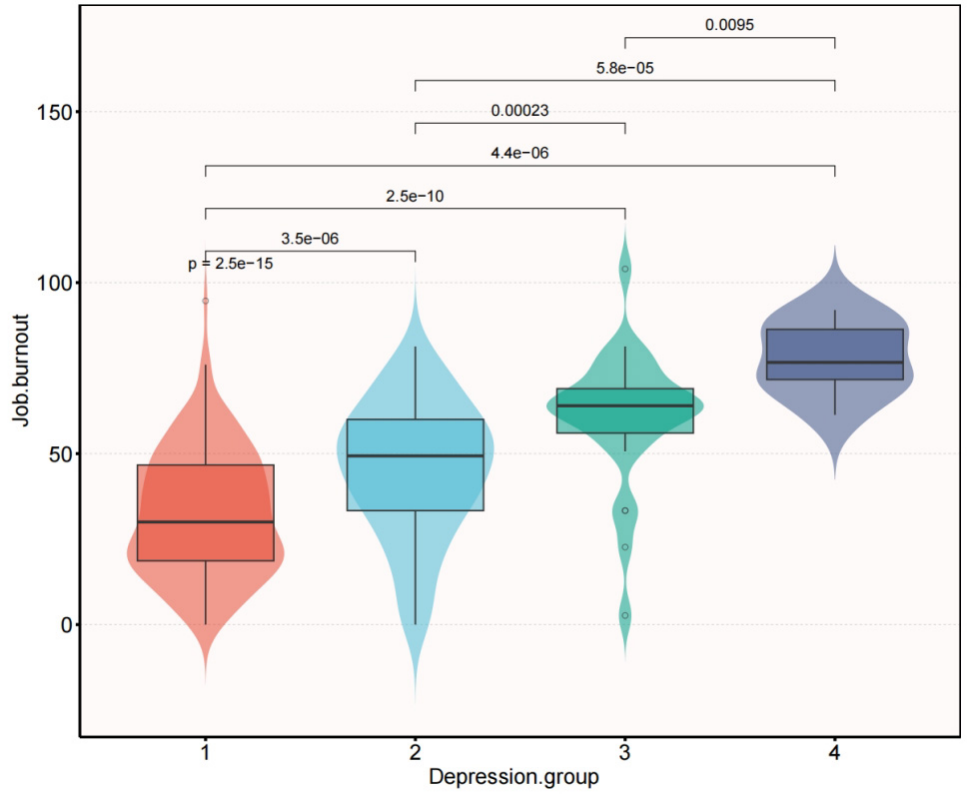
Boxplot of job burnout scores in dental nurses with different depression level.

Figure 1 shows the restricted cubic spline curve of depressive scores and the likelihood of job burnout in dental nurses. The results indicated a significant positive association between depressive scores and the risk of occupational burnout (overall *P* < 0.001), and the non-linearity test was not statistically significant (*P* = 0.461), suggesting a linear correlation between them. As depressive scores increased, the likelihood of reporting burnout symptoms displayed a sustained upward correlational trend, with a steeper rising gradient observed above the depressive score cutoff of 50.

#### 3.5.2 Boxplot presentation of job burnout among dental nurses with different depression levels

**Figure 2:**
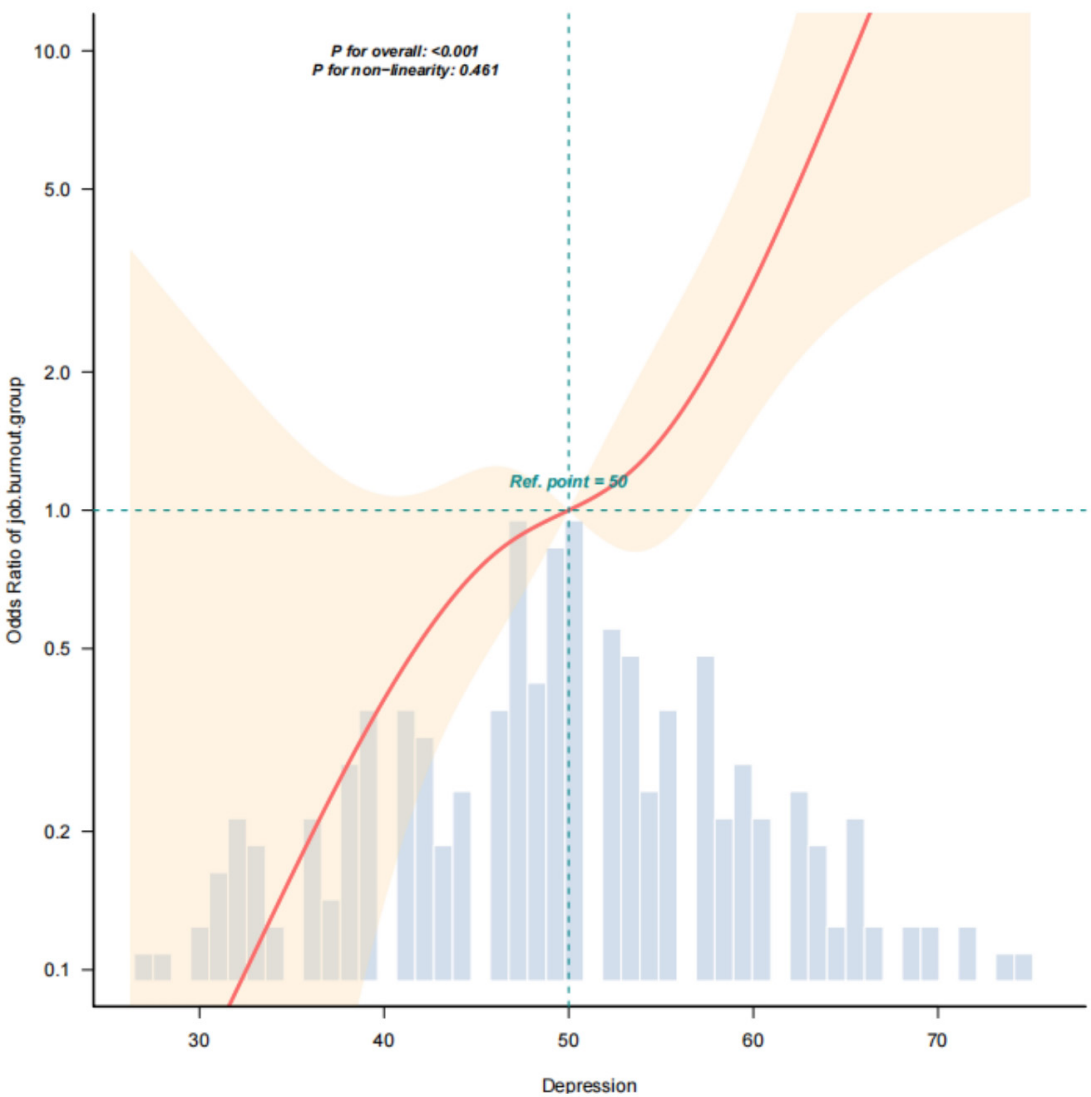
Curve fitting plot of the relationship between depression and job burnout in dental nurses.

Figure 2 shows violin boxplots of job burnout levels (continuous scale score here, dichotomous variable in Logistic regression analysis) in dental nurses across four depressive severity groups (no, mild, moderate, severe depression). The results indicated that job burnout levels increased progressively with depressive severity (all *P* < 0.05); the severe depression group had significantly higher median burnout levels than other groups, while the no depression group was the lowest, suggesting more severe depression correlates with higher job burnout in dental nurses.

## 4 Discussion

This study was a cross-sectional study of dental nurses at a stomatological hospital in Yunnan Province, China, which explored the status and associated factors of dental nurses' job burnout. We enrolled 261 dental nurses, and the incidence of job burnout in these participants was 33.7%, which is higher than that in Wang Jing's survey (28) of more than 3,000 medical workers in 11 provinces of China (26.9%). The possible reason for this difference lies in the fact that, on the one hand, this study only focused on a stomatological hospital in Yunnan Province, while Wang Jing's research involved multiple provinces, covered various medical institutions, and had a large sample size. There are significant differences in the medical environment across different provinces, as well as in the work content and stressors among different institution types (especially between stomatological hospitals and general/primary institutions), may partly explain the inconsistent burnout prevalence. On the other hand, our participants were dental nurses, while Wang Jing's research included dentists and dental nurses. Compared with dentists, dental nurses have a low social status. They have low job autonomy, lack a sense of participation in decision-making, and have obvious career development ceilings with limited promotion opportunities. Moreover, they are relatively low salary, which was correlated with elevated burnout symptoms. Previous studies have shown that compared with other medical occupations, nurses have a higher risk of job burnout, which also confirms this point. Nonetheless, we found a higher incidence of burnout among nurses in other medical departments. For instance, the rate is 42.5% among operating room nurses (29), 57.3% among emergency department nurses (30), 64.7% among ICU nurses (31), and 38.6% among pediatric nurses (high levels of burnout) (32). Two occupational features specific to dental nursing may help interpret this comparatively mild burnout burden in our cohort., In terms of working environment, dental diagnosis and treatment mainly focus on common and frequently occurring outpatient diseases, with relatively mild patient conditions and an extremely low proportion of critical illnesses. Additionally, the risks to their own safety and health (such as occupational exposure) during work are fewer. Meanwhile, the nurse-patient communication focuses on treatment cooperation and health guidance, resulting in more harmonious relationships and relatively less psychological pressure. With respect to workload, dental nurses have a more moderate work intensity, and the frequency of night shifts is significantly lower than that of other departments, with relatively regular work schedules and more sufficient physical and mental recovery. Collectively, these occupational characteristics are correlated with lower burnout prevalence among dental nurses in the current cross-sectional sample.

The results of binary logistic regression analysis showed that depression was an independent associated factor of job burnout among dental nurses (OR = 1.117, *P* < 0.001), dental nurses with higher levels of depression had a higher likelihood of experiencing job burnout, which is consistent with the research findings of Larysz (33) and Chohan (34). The boxplot results further intuitively presented this association: the median score of occupational burnout in dental nurses with depressive symptoms was significantly higher than that in the non-depressive group. This suggests that the association between depression and occupational burnout is not an accidental difference but a stable statistical correlation, providing visual evidence for the regression results. Meanwhile, the restricted cubic spline curve revealed the quantitative correlation pattern between the two—depression severity was linearly positively correlated with job burnout. When the depression score exceeded 50, the risk of occupational burnout showed an accelerating upward trend. This “dose-effect” indicates that the statistical linkage of depression on occupational burnout is not a simple “presence-absence” relationship but a cumulative effect that progresses with severity, further strengthening its clinical significance as a core associated factor.

This result can be explained by combining the work characteristics and psychological mechanisms of dental nurses. Although dental nurses do not face the same intense work environment as nurses in departments like the ICU, some still experience chronic high stress in their roles. For one thing, patients' fear of dental procedures, coupled with their high expectations for treatment outcomes, the close attention of family members to the safety and experience of patients (especially pediatric ones), and the irritable personalities of some patients, may trigger nurse-patient conflicts and elevate psychological burden. For another, the high demands for precision during four-handed dentistry can easily trigger persistent tension. These findings suggest that when individuals experience depressive moods, their tolerance threshold for such stressors decreases, making them more susceptible to burnout manifestations like emotional exhaustion and depersonalization. Depressive moods are often accompanied by negative cognitions (such as pessimism and hopelessness), difficulty concentrating, and impaired decision-making. These symptoms deplete an individual's psychological resources, making them more prone to feelings of fatigue, detachment (depersonalization), and reduced personal accomplishment when facing work pressures, showing a correlated tendency toward burnout symptoms. This also corroborates the perspective of the JD-R model: depressive mood, as a depletion of an individual's psychological resources, weakens their ability to cope with job demands, which displays a correlated relationship with elevated burnout symptoms (14). In conclusion, depressive mood among dental nurses serves as a crucial associated factors and intervention target for their job burnout. Safeguarding the mental health of dental nurses, preventing and intervening in depression, is a key link in enhancing their occupational well-being, ensuring the quality of care, and stabilizing the dental healthcare workforce. This requires the collective effort of individuals, teams, managers, and the broader healthcare system.

These results also demonstrates that occupational stress was an independent associated factor of job burnout among dental nurses (OR = 1.063, *P* < 0.001), which aligns with the findings of surveys by Li (35) and Hetzel-Riggin (36). Based on the JD-R model (14), occupational stress is essentially a core “job demand” in dental nursing practice—including high-precision requirements for clinical operations, communication pressure caused by patients' fear of treatment, and excessive workload. When “job resources” such as team support and professional training fail to match effectively, nurses will fall into an imbalanced state of “resource consumption > supplementation”, this state is statistically linked to elevated burnout manifestations such as emotional exhaustion and depersonalization via the energy depletion pathway proposed, which also confirms the intrinsic mechanism of occupational stress as a associated factors of burnout. Therefore, alleviating occupational burnout of dental nurses needs to start from the dual dimensions of balancing job demands and supplementing resources. Collaborative multi-faceted interventions that effectively block the pathway from stress to burnout can help safeguard the physical and mental health of dental nurses, maintain workforce stability, and ultimately provide patients with safe and high-quality care services.

Notably, univariate analysis revealed that social support, self-efficacy, and career identity were beneficial correlates associated with lower occupational burnout levels (*P* < 0.05). Although these factors did not enter the binary logistic regression model as independent associated factors, they still hold important practical reference value. The reason why these factors failed to become independent associated factors may be related to the limited sample size of this study, the interaction between variables (e.g., nurses with high self-efficacy tend to obtain more social support), or the interference of confounding factors. These favorable correlational associations observed in univariate analysis indicate that improving the social support level of dental nurses (e.g., departmental humanistic care, colleague mutual assistance) and enhancing professional self-efficacy and identity (e.g., specialized training, career development planning) may act as promising auxiliary intervention targets linked to milder burnout symptoms, providing supplementary evidence for formulating diversified burnout prevention and control strategies in the field of dental nursing.

In this study, the results of binary logistic regression showed that none of the sociodemographic factors were associated factors of job burnout, although some of them were statistically significant in the univariate analysis. Two potential explanations for this discrepancy are proposed on the one hand, variables such as age, weekly exercise time are essentially associated with burnout indirectly by influencing “occupational stress” or “depression” (e.g., “increasing age” may be accompanied by the accumulation of “occupational stress”). When these variables are incorporated into the regression model simultaneously, their weak correlational associations are masked by the robust correlational links of occupational stress and depression, and their independent roles are no longer statistically significant. On the other hand, Univariate analysis fails to exclude the interference of other variables, which may lead to “spurious correlations”, in contrast, regression analysis filters out such interference through multivariate control, retaining only the variables (occupational stress and depression) that display unique independent correlational associations with burnout outcomes.

In light of our findings, interventions should target both individual and organizational levels. At the individual level, addressing depressive symptoms through psychological support is crucial. At the organizational level, hospitals should optimize workloads and enhance job resources to mitigate occupational stress. Several interventions (e.g., mindfulness-based programs, yoga, organizational-level reforms) have been suggested to reduce burnout in other healthcare populations (33–36). However, none of these has been specifically evaluated in dental nurses, and their applicability to this population requires future research."

Ultimately, combating burnout requires a paradigm shift from reactive, individual-focused interventions to proactive, system-level investments. Viewing the well-being of dental nurses as a barometer for the health of the oral care system itself and merging systemic reform with value-based care is the first step toward building a more resilient and compassionate infrastructure.

This study has several limitations that should be acknowledged. First, the cross-sectional design only captures data at a single time point, which cannot clarify the temporal sequence between variables and precludes any causal inference; we can only interpret correlational associations rather than causal effects. Second, all outcomes and independent variables were collected via self-reported questionnaires, which may introduce recall bias and social desirability bias. Third, simultaneous collection of all survey items may lead to common-method bias that cannot be fully eliminated in this study. Fourth, participants were recruited from only one stomatological hospital using convenience sampling, which creates potential selection bias and restricts the external validity; the validity of these findings in other types and regions of dental institutions remains uncertain and requires further validation. Fifth, several unmeasured residual confounding factors (e.g., family stress, long-term occupational history) were not included in the regression model, which may partially interfere with the observed associations. In addition, although the sample size met the statistical requirements for multivariate logistic regression, larger multi-center samples are still needed to verify the results. For future research, mixed-method designs combining quantitative surveys and qualitative interviews are recommended to achieve more comprehensive triangulation of burnout-related factors among dental nurses.

## 5 Conclusion

This study focuses on job burnout among dental nurses, incorporates multiple psychosocial factors, and identifies the key influencing factors for job burnout in this population via binary logistic regression analysis. These findings underscore the imperative of prioritizing the mental health of dental nurses and implementing targeted interventions to mitigate their job burnout. An in-depth understanding of the influencing factors for job burnout can effectively reduce nurses' turnover rates, lower job vacancy rates, alleviate human resource shortages, and also play a pivotal role in enhancing nursing quality. Nursing quality is the direct manifestation and core objective of value-based nursing. Without burnout, specialized dental nurses can continuously improve nursing quality and create greater value. This study further pinpoints core influencing factors for job burnout, including depression and occupational stress. Therefore, potential interventions to alleviate burnout can be implemented at both organizational and individual levels: at the organizational level, a comprehensive and systemic nurse care program should be promoted to enhance dental nurses' social support, professional identity and self-efficacy, which in turn reduces their depression and occupational stress, fosters a harmonious working environment, further decreases the incidence of burnout and stimulates nurses' motivation and vitality; at the individual level, measures such as mindfulness training, meditation, yoga, and the like can be adopted.

## Data Availability

The raw data supporting the conclusions of this article will be made available by the authors, without undue reservation.
